# Marked response to dupilumab in an elderly patient with eosinophilic annular erythema

**DOI:** 10.1016/j.jdcr.2025.07.032

**Published:** 2025-08-30

**Authors:** Yoko Yamamoto, Hiroko Wakimoto, Rika Kikuchi, Kazutoshi Harada

**Affiliations:** a Department of Dermatology, Tokyo Medical University, Tokyo, Japan; b Miyabayashi Clinic, Tokyo, Japan

**Keywords:** dupilumab, elderly patient, eosinophil, eosinophilic annular erythema, suplatast tosilate

## Introduction

Eosinophilic annular erythema (EAE) is a relatively rare but recurrent disease characterized by annular erythema with eosinophilic infiltration. Herein, we report an elderly patient with EAE which had been poorly controlled for 20 years but which finally resolved after the administration of dupilumab, an interleukin (IL)-4 receptor α antagonist.

## Case report

**Fig 1:**
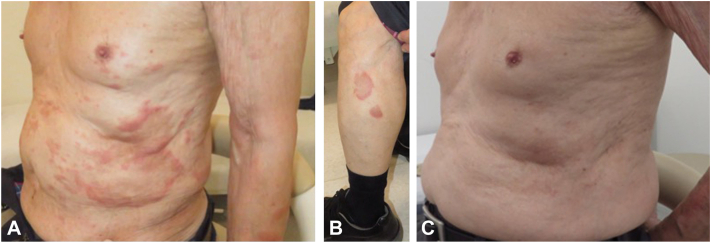
**A** and **B,** Multiple, mildly pruritic, annular erythema with peripheral infiltration on the trunk and limbs. **C,** By 2 weeks after the start of dupilumab therapy, all the skin rashes had improved significantly.

**Fig 2:**
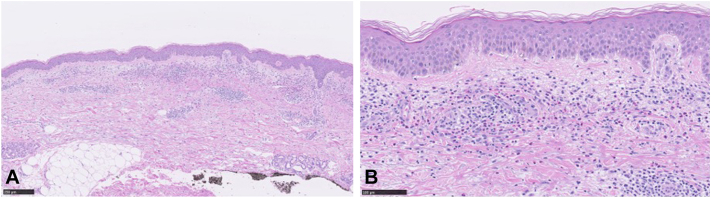
**A,** Infiltration of inflammatory cells consisting mainly of eosinophils around the vascular area of the superficial dermis (HE; bar: 250 μm). **B,** Eosinophilic infiltration limited to the dermis (HE; bar: 100 μm).

An 87-year-old, male, Japanese patient presented with recurrent erythema on the trunk and extremities of 20 years’ duration. The lesions responded to a topical steroid but recurred multiple times. His medical history included hypertension, prostatic enlargement, and allergic rhinitis. A physical examination revealed multiple, annular, mildly pruritic erythemas without scaling on the trunk and limbs (Fig 1, *A* and *B*). A laboratory examination revealed mild elevation of thymus and activation-regulated chemokine. No increase in the eosinophil count or anti-BP180 antibody was detected. Histological examination of an erythema on the right thigh demonstrated eosinophil-predominant infiltration of inflammatory cells around the vessels of the superficial dermis (Fig 2). Necrotizing vasculitis and flame figures were denied. Immunofluorescence found no immunoglobulin or complement deposition on the basement membrane or perivascular areas. Based on these clinical, pathological, and direct immunofluorescence findings, EAE was diagnosed.

Initially, suplatast tosilate, an inhibitor of production of type 2 cytokines, such as IL-4 and IL-5 from Th2 cells, was prescribed but produced only a partial response. After suplatast tosilate administration, the patient received subcutaneous dupilumab 600 mg on day 0 followed by 300 mg every 2 weeks. Interestingly, all the lesions improved significantly in 2 weeks (Fig 1, *C*). After 8 doses, the lesions were under control, and the administration interval was lengthened to every 3 weeks. No recurrence of the lesions was thereafter observed.

## Discussion

EAE is a rare, cutaneous condition first described in 1981.^1^ There are, as of yet, no predefined diagnostic criteria for EAE. Żychowska et al proposed the following criteria: (1) presence of annular, erythematous lesions characterized by a chronic, relapsing, and remitting course; (2) absence of blood eosinophilia (in the majority of cases); (3) histopathological findings of dense, perivascular infiltrates composed of numerous eosinophils and the absence of flame figures (except in long-lasting lesions); and (4) exclusion of other conditions (mainly Wells syndrome, urticaria, erythema annulare centrifugum, and lupus erythematosus).^2^ EAE was diagnosed in the present patient because all the above criteria were met.

EAE develops from type-2 inflammation. Thymus and activation-regulated chemokine induces eosinophilic infiltration in the dermis, and IL-5 induces the degranulation of the eosinophils, prolonging their survival.^3^ Although various treatments, including systemic corticosteroids, antimalarials, cyclosporin A, dapsone, indomethacin, nicotinamide, methotrexate, mepolizumab, suplatast tosilate, benralizumab, and dupilumab, have been tried to date, there is still no established treatment regimen for EAE. Long-term, oral steroid administration is effective but difficult to administer to elderly patients owing to the adverse effects. Suplatast tosilate, an inhibitor of type-2 inflammation, was not fully effective in the present patient; thus, dupilumab was additionally administered, resulting in a significant improvement in the skin symptoms. Both suplatast tosilate and dupilumab are reportedly effective against EAE.^4^^,^^5^ Why dupilumab elicited a favorable treatment response in the present patient while suplatast tosilate did not remains unknown, but the direct blocking of IL-4/IL13-signaling by the former may have contributed to controlling the disease.

A previous study reported that case of a 14-year-old, female patient was successfully treated for EAE using dupilumab.^4^ The present report described the use of dupilumab in an elderly patient with EAE. Its safety for elderly patients may make dupilumab a promising treatment for EAE, but further study is needed for a definitive conclusion.

## Conflicts of interest

None disclosed.
